# Adhesion of Two New Glass Fiber Post Systems Cemented with Self-Adhesive Resin Cements

**DOI:** 10.3390/dj7030080

**Published:** 2019-08-01

**Authors:** Esin Özlek, Prasanna Neelakantan, Jukka Pekka Matinlinna, Sema Belli, Mehmet Ugur, Idrıs Kavut

**Affiliations:** 1 Department of Endodontics, Faculty of Dentistry, The University of Van Yuzuncu Yil, Van 65090, Turkey; 2 Discipline of Endodontology, Faculty of Dentistry, The University of Hong Kong, Hong Kong, China; 3 Dental Materials Science, Discipline of Applied Oral Sciences, Faculty of Dentistry, The University of Hong Kong, Hong Kong, China; 4 Department of Endodontics, Faculty of Dentistry, The University of Selcuk, Konya 42250, Turkey; 5 Department of Prosthodontics, Faculty of Dentistry, The University of Van Yuzuncu Yil, Van 65090, Turkey

**Keywords:** adhesion, glass fiber post, resin cement, self-adhesive, push out, bond strength

## Abstract

The aim of this in vitro study was to evaluate the adhesion strength of two new fiber post systems (FiberSite™ Post and Cytec™ Blanco Post) cemented with two different adhesive resin cements (Panavia™ SA and Maxcem™ Elite). Root canals of sixty extracted human mandibular premolars were prepared using ProTaper Universal™ rotary files (Dentsply Sirona Endodontics, York, PA, USA). The root canals were irrigated with 5.25% sodium hypochlorite (NaOCl) during instrumentation. After root canal preparation, the canals were irrigated with 2 mL of 17% EDTA (1 min), followed by 2 mL of 5.25% (5 min) NaOCI, and 2 mL saline. The root canals were dried with paper points and divided randomly into two study groups (*n* = 30) according to the type of post system: Group 1, FiberSite™ Post (MegaDental, Partanna, Italy); and group 2, Cytec™ Blanco Post (Hahnenkratt, Königsbach-Stein, Germany), with one of the two adhesive resin cements: Subgroup A, Panavia™ SA Cement Plus Automix (Kuraray, Osaka, Japan); subgroup B, Maxcem™ Elite (Kerr, Orange, CA, USA). Following thermocycling, the adhesion strength was evaluated using the push-out adhesion (bond) strength test. Fractographic analysis was performed using stereomicroscope. The data were analyzed using two-way analysis of variance (*p* = 0.05). The adhesion strength values of both the posts were significantly higher when cemented with subgroup B (Maxcem™ Elite). The highest adhesion strength value was demonstrated by group 1B (FiberSite™ post cemented with Maxcem™ Elite cement). The type of post did not have a significant impact on the bond strength values for either cement material.

## 1. Introduction

Root canal treated teeth with extensive loss of tooth structure oftentimes need a synthetic post to retain the core that will be built up. Posts may be categorized into metallic, fiber-based, and ceramic [1]. Metallic posts have been the longest standing in terms of availability. However, clinical difficulties in terms of preparation time, aesthetics, and the potential mismatch in the elastic modulus of these posts compared to the root dentin, have resulted in the search and development of alternative post systems.

In contemporary dental practice and adhesive dentistry, one of the most commonly used post systems is the E-glass fiber-reinforced composite resin (FRC) post [2]. E-glass fibers are silanized for durability and adhesion with the resinous matrix [3]. The elastic modulus of these posts is closer to dentin than metallic posts, thereby mitigating the risk of vertical fractures of root canal treated teeth. Furthermore, adhesion and the aesthetics of these posts is an added advantage when used in the anterior teeth [4]. The retention of the fiber posts in the root canal is influenced by various factors such as the type of the post, length, shape, surface properties of the post, adaptation of the post to the prepared cavity, and the type of adhesive agent used [4]. Recently, several modifications have been introduced into FRC posts. One interesting strategy has been designing an E-glass fiber post with a pre-shaped abutment. This post (FiberSite™, Mega Dental, Italy) is an anatomic post. However, it remains unclear whether the bond strength of this novel post design differs from that of other FRC posts. 

FRC posts are cemented to the root canal dentin using adhesive resin cements, which require dentin surface treatment in the form of etching/conditioning and application of a dentin bonding agent. That said, self-adhesive resin composite cements were introduced with the claim of overcoming the potential problems related with the dentin status after etching and rinsing, as well as to enhance predictable cementation of fiber posts, in addition to decreasing the treatment time [5].

There are many studies in the literature regarding the choice of material and the luting process that may affect the bond strength of the FRC posts. Studies have reported that the use of self-adhesive resin cements increases the bond strength of fiber posts. The aim of this in vitro study was to evaluate the adhesion strength of two new fiber post systems (FiberSite™ Post and Cytec™ Blanco Post), cemented with two different adhesive resin cements (Panavia™ SA and Maxcem™ Elite) [6,7]. Bond strength was measured by using the push-out bond strength test. The null hypothesis was that neither the post type, nor the type of resin cement, influenced the adhesion strength of the posts to dentin. 

## 2. Materials and Methods

### 2.1. Specimen Preparation

This study used freshly extracted, single-rooted mandibular human premolars (*n* = 60), which were extracted for orthodontic reasons. These teeth had completely formed roots and closed apices, with no cracks or structural anomalies. The presence of a single root canal was confirmed by taking radiographs in two angulations (mesiodistal and labiolingual). According to ISO standards, the teeth to be used for the test were used within 1–6 months after tooth extraction. The teeth were kept in the refrigerator at +4 °C in distilled water or in 0.5% chloramine-T solution for a maximum of 1 week and then at +4 °C in distilled water. Human teeth collected from individuals between the ages of 16–40 were used. The disinfection and storage conditions of the teeth used in our study were performed according to ISO standards [8]. The study protocol was approved by the Non-Interventional Clinic Research Ethics Board of Yuzuncu Yil University, Van, Turkey (Approval number 09) on 20 June 2017.

The root surfaces of the teeth were cleaned and the specimens were then decoronated with a slow-speed diamond saw (Isomet 1000, Buehler, Lake Bluff, IL, USA) under water cooling. Roots with standardized lengths of 10 mm were thus obtained. The working length was established visually by subtracting 1 mm from of an ISO size 15 file placed at the apical foramen. The root canals were instrumented with rotary nickel titanium instruments (ProTaper™ Universal, Dentsply Maillefer, Ballaigues, Switzerland) up to F3. During instrumentation, root canals were irrigated with 2 mL of 5.25% sodium hypochlorite (Imicrly™, Konya, Turkey) using a 31G side-vented needle, placed 1 mm short of the working length. Following instrumentation, the root canals were irrigated with 2 mL of 17% EDTA (Imident™ Med., Konya, Turkey) for 2 min, to remove the smear layer. The root canals were then rinsed with 5 mL of distilled water and dried with paper points.

### 2.2. Cementation of Posts

The specimens were randomly distributed into two study groups (*n* = 30) using a computer program (www.random.org), based on the post system used:

Group 1 FiberSite™ Post system (MegaDental, Partanna, Italy);
Group 2 Cytec™ Blanco Post (Hahnenkratt, Königsbach-Stein, Germany).

Posts with a diameter of 1.6 mm for Cytec Blanco and 1.8 mm for FiberSite, and 10 mm in depth, were opened with the drills coming out of the kit. Specimens in both groups were divided into two subgroups based on the resin cement used (*n* = 15): Subgroup A, Panavia™ SA Plus Automix (Kuraray, Osaka, Japan); and subgroup B, Maxcem™ Elite Cement (Kerr, Orange, CA, USA). In all the specimens, the cements were injected into the root canals, after which the posts, covered with the resin cement, were seated to the full depth of the root canal space. The post was then light-cured for 20 s. 

### 2.3. Thermal Cycling (Artificial Ageing)

After 72 h of storage in an incubator at 37 °C, the specimens were thermocycled in water between 5 and 55 °C for 10,000 cycles, with 20 s dwell time (Termal Siklus, Dental Teknik, Konya, Turkey). The teeth were then embedded in epoxy resin using a split-ring copper mold. Using an Isomet™ saw (Buehler, Lake Bluff, IL, USA) under water cooling, 8 sections were obtained from each tooth. Each section was 1.0 mm ± 0.1 mm thick, and this was measured using a digital caliper to 0.04 mm accuracy (Mitutoyo, Tokyo, Japan). The coronal surface of each specimen was indicated using a permanent marker to allow identification during the loading for push-out testing. 

#### 2.3.1. Bond Strength Test

The bond strength was determined using the push-out test based on a methodology published earlier [6]. Briefly, stainless steel pluggers of diameter 0.9 mm were used in a universal testing machine (LIoyd™ LRX-plus; LIoyd Instruments, Fareham, UK) at a crosshead speed of 1.0 mm/min to apply push-out force in the apico-coronal direction. A sudden drop in load deflection indicated bond failure. Push-out bond strength (MPa) was calculated based on the formula reported earlier [9]: Push-out bond strength (MPa) = N/A; where N = maximum load (N), and A = adhesion area of root canal filling (mm^2^). The bonding surface area of each section was calculated as: [π (r_1_ + r_2_)] × [(r_1_ − r_2_)^2^ + h_2_]^1/2^; where π is the constant 3.14, r_1_ and r_2_ are the smaller and larger radii, respectively, and h is the thickness of the section (mm).

#### 2.3.2. Failure Mode Analysis

After the push-out test, all samples were examined under stereomicroscope (Olympus SZ X7; Leica, Wetzlar, Germany) at 56× magnification to determine the mode of failure and classified into: (i) Adhesive failure between the cement and root-dentin; (ii) adhesive failure between the fiber post and cement; (iii) mixed failure; (iv) cohesive failure within the cement, dentin, or fiber post. In these samples, random allocation and allocation concealment was made by random selection, and 8 samples reanalyzed under SEM at 300× magnification.

#### 2.3.3. Data Analysis

As the data were not distributed normally, suitable transformation methods were tried and the data obtained as a result of the square root transformation were determined to be in normal distribution. The data were analyzed using two-way analysis of variance. The significance level was set at 0.05 for all the statistical analyses.

## 3. Results

### 3.1. Bond Strength

Two-way analysis of variance was performed using the square root data and the results were presented in Table 1. The values presented in the descriptive statistics table are square root values and the squares should be taken for the original values (Table 2).

According to the two-way analysis of variance, the main effect of the group was not statistically significant (*p* = 0.127). The mean value for FiberSite™ was 1.335 MPa, while the average value for Cytec™ Blanco was 1.267 MPa. Subgroup main effect was statistically significant (*p* < 0.001). While the average value for Maxcem™ Elite was 1.413 MPa, the average value for Panavia™ SA Plus was 1.188 MPa. The mean value obtained in the Maxcem™ Elite subgroup was higher. Group and subgroup interaction was not statistically significant (*p* = 0.591).

### 3.2. Failure Mode

**Figure 1:**
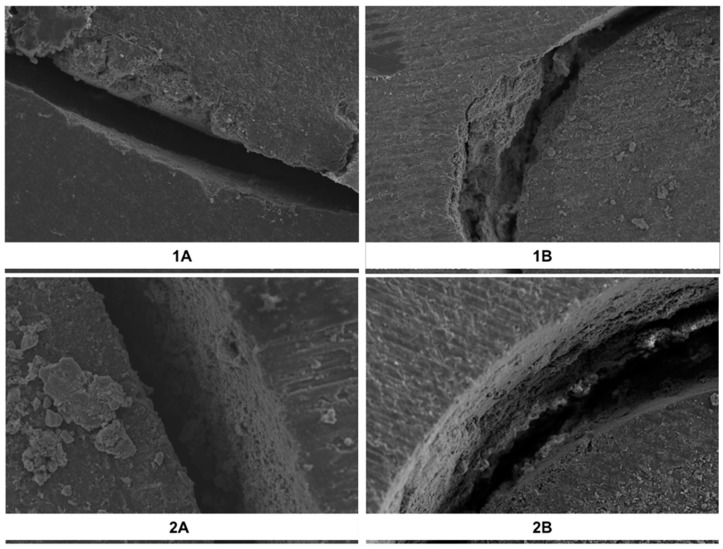
Representative SEM images of the interface demonstrating the failure modes. The figure shows a sample with adhesive failure in group (**1A**), mixed failure from group (1B), adhesive failure from group (**2A**), and cohesive failure from group (**2B**).

The failure analysis results of the samples have been summarized (Table 3). Representative SEM images demonstrating the failure modes are shown (Figure 1). The dominant failure mode in all of the groups was adhesive (between cement and dentin). Less than 2% of the samples demonstrated cohesive failure within the resin composite cement. 

## 4. Discussion

This laboratory study evaluated the bond strength of two resin composite cements used to cement two types of new E-glass fiber posts inside root canals. The results suggested that the adhesion strength values did not depend on the post type. However, the resin composite cement type demonstrated significant differences in the bond strength for both the post types. That said, the null hypothesis needs to be partially rejected. 

Resin composite cements in dentistry have dual roles: Durable adhesion and increased fracture toughness. Both of the resin composite cements used in the current study were the so-called self-etching, self-adhesive cements. The automix versions for both cements were used in this study. It has been reported that automixed resin composite cements demonstrate significantly higher mechanical properties (in particular, compressive strength) than their hand-mixed variants [10]. The use of self-etching and self-adhesive resin composite cements is clinically advantageous as it shortens the chairside time, and also eliminates the guesswork on the substrate characteristics, specifically in terms of moisture [11]. In the etch and rinse adhesive systems, excessive roughening of the dentin may result in incomplete resin infiltration and weakening of the connection. Due to the simultaneous acidification and resin infiltration of self-etch resin cements, the possibility of incomplete infiltration is low and the wetting and dentin connection with acidic monomers in cement material increases [12,13]. Self-etching and self-adhesive resin composite cements do not require any specific surface pre-treatment (conditioning) as the resinous matrix is composed of phosphoric acid and/or carboxylic acid methacrylate monomers [14]. Since these cements do not form a hybrid layer, adhesion strengths of this category of cements to dentin are significantly lower than for those adhesive resin composite cements where substrate pre-treatment is required [15,16,17]. It is noteworthy that no study to date has compared and contrasted the two self-etching and self-adhesive cements that were evaluated in the current study, to cement two types of E-glass fiber posts. Many studies have been conducted to evaluate the factors affecting the retention of posts, and to increase post-resin cement connectivity. It has been shown that various factors such as length, diameter, shape, surface structure, type of post, thickness of cement layer between post-dentine, applied surface treatment, and roughness of root canal dentin surface affect the retention of post. Additionally, the type of resin composite cement used is important in increasing post retention (durable adhesion) and higher fracture toughness of endodontically treated teeth [7,18,19,20,21].

There are some methodological aspects of the current study that warrant further discussion. The E-glass posts were cemented inside root canals that were not previously filled with gutta-percha and resin cement. In the endodontic clinical situation, a post space is prepared after removing the root filling materials in order to cement a post. However, when bond strength of resin composite cements is being studied, the remnant root canal sealer on the walls will induce a confounding factor. Self-adhesive resin cements based on dual adhesive (bifunctional) monomers such as 10-methacryloyloxydecyl dihydrogen phosphate (10-MDP) bind chemically to the calcium atoms in the dentin, thereby demonstrating durable chemical (ionic) bond integrity [22]. This is possible because of its methacrylate and organophosphate groups, separated by the 10 CH_2_ unit long linker part. There is evidence to show that the most important reason for poor bond strength of resin composite cements/fiber posts to dentin is the difficulty in removing the smear layer from the root canal walls [23,24,25]. There are recent interesting suggestions of dentin conditioning with boric acid, H_3_BO_3_ [26] to remove the smear layer. On the other hand, the use of synthetic hydroxyapatite might be helpful for enhancing chemical bonding [27] when using self-adhesive resin composite cements. In addition, it may be thought that the poor bond strength may be due to the high C factor, which is the ratio of the bonded surfaces to the unbonded surfaces. During polymerization, there is the possibility of separation of the bonded regions due to insufficient flow and stress as the unbound surface area becomes smaller [28].

Nevertheless, dentin is inherently moist, containing approximately 10% water by weight. Resin composite cements that are bonded to dentin absorb some of this water, and this further influences the service life of fiber posts. It is indeed understandable that such diffusion of water plays an important role in the chemical and thermal fatigue processes of endodontic posts [29,30,31,32]. For this reason, and to simulate the environment (at least in some extent), artificial aging is widely used and accepted in the testing of dental materials. This study used thermocycling to simulate ageing. While the suggested regimen for thermocycling usually ranges between 3000–10,000 cycles at 5–55 °C, we chose 10,000 cycles as it represents about one year of service [33]. A 37% reduction in bond strength of fiber posts has been reported after thermal cycling [2,31,32,33,34,35]. Panavia™ SA contains MDP in resin cement and Maxcem™ Elite resin cement contains 2-hydroxyethyl methacrylate (HEMA) and glycerol 1,3-dimethacrylate (GDM) monomer. The water absorbed by these hydrophilic resin monomers reduces the mechanical strength of the adhesive, and can adversely affect the long-term stability of the resin-dentin connection [36]. In many studies in the literature, Panavia™ SA resin cements showed higher bond strength values than Maxcem™ Elite resin cements [6,7,13,30]. These results contradict our study. However, in all of the studies, the groups were tested immediately and the long-term effect was not compared. We think that the difference between the studies can be caused by the difference between the water absorption potentials of different monomers in the resin cements and the short- and long-term effects of these cements. We should also note that among resin cement differences, this may not be clinically important. Further clinical studies are needed.

Today, adhesion of endodontic materials to root dentin can be assessed by different standardized testing methods such as traditional shear and push-out tests. The push-out test (or dislocation resistance test) has been reported to be a reliable and reproducible test for assessing adhesion to root dentin [37]. The push-out test allows for better and deeper evaluation of adhesion strength than the traditional shear bond strength test as in endodontic research, the fractures are parallel to the dentin-resin bonding surface and thereby similar to the clinical situation [38]. It has also been reported that the push-out test may, in fact, be a test of frictional resistance that leads to misinterpretation of results. However, that is disputable and, on the other hand, it has been demonstrated that using specimens that are 1 mm in thickness overrides this risk [36]. There is still no definite evidence about the size of the fiber posts in the root canal in the literature. Hunter et al. reported that the concentration of the stress was concentrated in the apical region by the length of the posterior length extending to the middle [39]. Fernandes and Dessai reported that the use of taller fiber posts showed higher fracture strength [40]. Adanır and Belli stated that short post placement should be avoided in order to avoid clinical failure [41]. In contrast, Cechin et al. obtained similar fracture strengths with the postings of 8 mm and 12 mm in length, and were suspicious of the tall post placement requiring more preparation [42]. Due to these contradictory findings in the literature, the fiber posts were placed in the 10 mm long root canal in order to ensure standardization in all test groups. In the current study, we used sections with a thickness of 1 mm to reduce potential friction-induced inhomogeneous stress distribution. Furthermore, we plan to continue these studies by looking into the adhesion strength of these resin composite cements according to certain different strategies of post surface and dentin surface treatment concepts.

## 5. Conclusions

Under the conditions of this in vitro experiment, Maxcem™ Elite self-adhesive resin composite cement demonstrated significantly higher adhesion strength than Panavia™ SA cement, independent of the post system used.

## Figures and Tables

**Table 1:** The two-way ANOVA for the post systems, the resin cements, and the interaction terms, according the push-out bond strength (MPa).

Source	Type III Sum of Squares	df	Mean Square	F	Sig.
Group (the post system used)	0.416	1	0.416	2.335	0.127
Subgroup (the resin cements)	4.565	1	4.565	25.645	<0.001
Group * Subgroup	0.052	1	0.052	0.290	0.591

* Interaction between the post systems and the resin cements.

**Table 2:** Push-out bond strength (MPa, means ± standard deviations) of each group.

Resin Cements	FiberSite™	Cytec™ Blanco	Total
Maxcem™ Elite	1.435 ± 0.426	1.391 ± 0.390	1.413 ± 0.408
Panavia™ SA Plus	1.234 ± 0.405	1.142 ± 0.463	1.188 ± 0.436
Total	1.335 ± 0.427	1.267 ± 0.445	1.301 ± 0.436

**Table 3:** Failure mode (%) for each group.

Group	Adhesive	Mixed	Cohesive
1: FiberSite Post system			
Subgroup A	85	0	15
Subgroup B	89.16	0.84	10
2: Cytec Blanco Post			
Subgroup A	83.34	0	16.66
Subgroup B	88.34	0	11.66
